# Laterally wedged insoles in knee osteoarthritis: do biomechanical effects decline after one month of wear?

**DOI:** 10.1186/1471-2474-10-146

**Published:** 2009-11-25

**Authors:** Rana S Hinman, Kelly Ann Bowles, Kim L Bennell

**Affiliations:** 1 Centre for Health, Exercise and Sports Medicine, School of Physiotherapy, The University of Melbourne, Victoria, Australia

## Abstract

**Objective:**

This study aimed to determine whether the effect of laterally wedged insoles on the adduction moment in knee osteoarthritis (OA) declined after one month of wear, and whether higher reported use of insoles was associated with a reduced effect on the adduction moment at one month.

**Methods:**

Twenty people with medial compartment OA underwent gait analysis in their own shoes wearing i) no insoles and; ii) insoles wedged laterally 5° in random order. Testing occurred at baseline and after one month of use of the insoles. Participants recorded daily use of insoles in a log-book. Outcomes were the first and second peak external knee adduction moment and the adduction angular impulse, compared across conditions and time with repeated measures general linear models. Correlations were obtained between total insole use and change in gait parameters with used insoles at one month, and change scores were compared between high and low users of insoles using general linear models.

**Results:**

There was a significant main effect for condition, whereby insoles significantly reduced the adduction moment (all p < 0.001). However there was no significant main effect for time, nor was an interaction effect evident. No significant associations were observed between total insole use and change in gait parameters with used insoles at one month, nor was there a difference in effectiveness of insoles between high and low users of the insoles at this time.

**Conclusion:**

Effects of laterally wedged insoles on the adduction moment do not appear to decline after one month of continuous use, suggesting that significant wedge degradation does not occur over the short-term.

## Background

Knee osteoarthritis (OA) is a prevalent musculoskeletal condition [1] that is associated with considerable pain, disability and loss of quality of life, particularly amongst the elderly. With the exception of costly joint arthroplasty typically reserved for end-stage disease, there is no cure for OA. Accordingly, there is an urgent need for conservative treatment strategies that not only alleviate symptoms of OA but also reduce the risk of disease progression over time.

Increased joint loads during walking have been implicated in disease pathogenesis [2], although the precise aetiology of OA remains unknown. The external knee adduction moment is an indicator of dynamic load borne across the medial tibiofemoral joint compartment and has been shown to predict the risk of OA progression in this compartment [3]. As a result, and combined with the fact that medial compartment OA is common [4], research is presently focussed on the development and evaluation of interventions that may reduce the peak adduction moment, with the ultimate view of reducing knee symptoms and the risk of medial compartment OA progression.

Laterally wedged insoles are orthotic devices placed within the shoes that have been recommended to manage medial knee OA [5,6]. Lateral wedges are hypothesised to reduce the moment arm of the ground reaction force vector relative to the knee joint centre during walking [7], and most biomechanical analyses demonstrate that lateral wedges can reduce the peak adduction moment in patients with knee OA by approximately 5-10% [7-10]. However most biomechanical studies have only evaluated the *immediate *within-session effect of lateral wedges in knee OA, providing no information as to whether the biomechanical effects of insoles decline over time. This is possible if the insoles were to compress or degrade with continued daily use.

It is particularly important to understand whether the biomechanical effects of wedged insoles decline over time given that longitudinal clinical trials evaluating their symptomatic effectiveness over 6 weeks [11], 6 months [12], 1 year [13] and 2 years [14] demonstrate no significant improvement in knee pain compared to control interventions. It may be that insoles need to be replaced quite frequently in order to maintain their wedged structure and sustain their biomechanical effect and thereby instigate improvements in knee pain. Although typically made of high density materials, it is possible that the insoles may compress over time, resulting in reduced wedging and rendering them less effective mechanically. This is particularly likely in patients with knee OA given their propensity for being overweight or obese and the need for prolonged and continuous use of the insole whilst weightbearing given the chronicity of the disease. Thus the aims of this study were to determine whether the beneficial effect of laterally wedged insoles on the adduction moment declined over one month of wear, and whether higher reported use was associated with a reduced effect on the adduction moment at one month. It was hypothesised that after one month of wear, the laterally wedged insoles would be less effective at reducing the knee adduction moment.

## Methods

### Participants

Participants in this study comprised a subset of people participating in an ongoing randomised controlled trial of lateral wedges [15]. Twenty community volunteers aged over 50 years with medial compartment knee OA were recruited by advertisement. Selection criteria were necessarily based on those used for the larger randomised controlled trial. Participants were included if they reported knee pain on most days of the previous month and demonstrated medial tibiofemoral osteophytes on x-ray [16]. Other inclusion criteria were an average knee pain >3 on an 11-point Likert scale when walking. Exclusion criteria included questionable or severe radiographic disease (Kellgren & Lawrence Grade 1 or 4); valgus knee alignment >185° on a standardised standing knee x-ray (as lateral wedges are unlikely to benefit such individuals); use of a gait aid; lateral tibiofemoral compartment joint space narrowing greater than medial; body mass index ≥ 36 kg/m^2^; hip or knee replacement; knee surgery or injection (past 6 months); use of insoles or foot orthotics (past 6 months), foot or ankle problem precluding use of insoles and; footwear incompatible with insoles.

In this study, only the symptomatic knee was tested. In the case of participants with bilateral symptoms, the more symptomatic knee was deemed the test limb. The University of Melbourne Human Research Ethics Committee approved the study and all participants provided written informed consent.

### Protocol

Participants underwent baseline gait analysis in their own usual footwear and whilst wearing a new pair of laterally wedged insoles. Testing occurred in randomised order. All participants were then given the insoles to take home and instructed to wear them everyday as tolerated. In order to maximise compliance, participants were permitted to transfer the insoles between shoes. After one month, participants returned for repeat gait analysis, both with and without their own worn pair of insoles inside the shoes. Testing occurred in the same randomised order and with the same shoes as used at baseline to ensure that any changes in the knee adduction moment were not due to differences in footwear [17].

### Laterally wedged insoles

**Figure 1:**
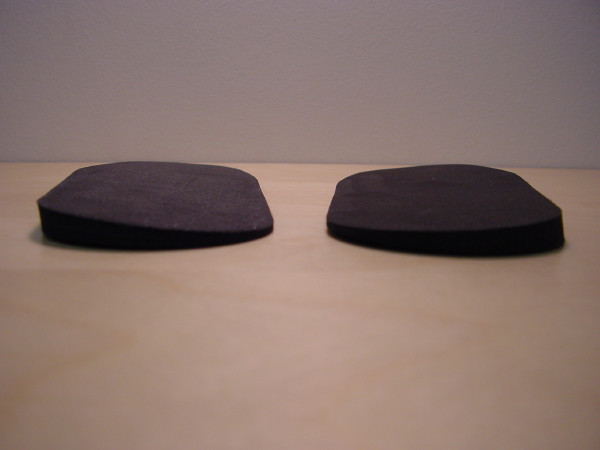
**The laterally wedged insoles**.

Standardised non-customised laterally wedged insoles were evaluated. Insoles were made of high density ethyl vinyl acetate, were wedged approximately 5° (as greater wedging is associated with foot discomfort [8]) and were worn bilaterally inside the participant's own shoes. The insoles were wedged along the lateral edge of the entire length of the foot (Figure 1). A Shore Durometer type A reading was taken on 20 samples of the wedge material from different batches of material used for the larger clinical trial and the mean hardness was found to be 57.5 (+/- 2.5). Participants were instructed to commence wearing the insoles for one hour, thereafter increasing use by one hour per day until wearing them full-time. Compliance was assessed by means of a log-book, where participants recorded the number of hours per day that the insoles were worn.

### Gait analysis

A Vicon motion analysis system with eight M2 CMOS cameras (1280 × 1024) operating at 120 Hz (Vicon, Oxford, UK) was used to measure the external knee joint adduction moment. The standard Plug-in-Gait marker set was used (anterior superior iliac spine, posterior superior iliac spine, mid-lateral thigh, lateral knee joint, lateral shank, lateral malleolus, on the shoe over the second metatarsal head and over the posterior calcaneus). Additional medial knee and ankle markers were used during the single static standing trial to determine tibial torsion. This was then used to compute the rotation of the shank segment marker plane required for placement of the ankle joint centre along a line between the malleoli in the dynamic trial data. Individual markers remained in situ throughout all test conditions.

Ground reaction forces were measured by two 0R6-6-2000 force plates (Advanced Mechanical Technology Inc., Watertown, MA) embedded in the floor at the midpoint of a 10 m walkway at 1080 Hz, in synchrony with the cameras. Participants walked at their usual comfortable pace and data were collected from 5 trials for each condition. Participants were not informed about the embedded force plates to prevent them "targeting" the plates and thus inadvertently altering their gait pattern. Several practice trials ensured that participants walked naturally and landed the whole foot of the test limb on the force plate. Walking speed was monitored by two photoelectric beams and verbal feedback ensured that speed during subsequent conditions and test sessions varied not more than 10% from the average speed of the first.

Joint moments were calculated via inverse dynamics (Vicon Plug-In-Gait v1.9). The knee adduction moment was normalised for body weight and height and reported in Nm/BW*HT% [3]. The dependent variables of interest were the external peak adduction moment in the first half of stance (first peak) and the external peak adduction moment in the second half of stance (second peak). The positive knee adduction angular impulse was also calculated (Nm.s/BW*HT%). The value of this measure is equivalent to the positive area under the adduction moment-time graph. This measure incorporates both the mean magnitude of the (positive) moment and the time for which it is imposed on the knee. Previous research has suggested that this measure may be a useful parameter in understanding gait patterns in sufferers of OA, complementing the more traditionally used peak knee adduction moment, as it accounts for more confounding factors of the disease including a decreased gait speed [18]. All variables were averaged over the 5 trials for each walking condition.

### Other measures

Radiographic disease severity at baseline was assessed using the Kellgren and Lawrence system [19]. The Western Ontario and McMaster Universities (WOMAC) Osteoarthritis Index assessed pain (score range 0-20, higher scores indicating worse pain) and physical function (score range 0-68, higher scores indicating worse function) at baseline only [20].

### Statistical analysis

Statistical analyses were performed using SPSS software (Norusis/SPSS Inc. Chicago, Illinois, USA) and an alpha level of 0.05. Adduction moment and walking speed data were evaluated using repeated measures general linear models, to determine the main effect for condition (insoles versus no insoles), time (baseline versus follow-up) and their interaction effect. Total insole use was determined by summing the number of hours insoles were worn over the 4-week period for each participant. One participant failed to record usage in week 4, and in this case, the mean of the previous three weeks was recorded as usage for that week. Relationships between total insole use and change in adduction moment parameters with insoles at follow-up were determined using Pearson r correlation coefficients. Finally, the cohort was dichotomised according to median total insole use after 4 weeks as either high-use (>222 hours/week) or low-use (≤222 hours/week). Change in adduction moment parameters with insoles at follow-up (calculated by subtracting the parameter measured with no insoles from the parameter measured when wearing insoles) was compared between these two groups using general linear models and adjusting for appropriate covariates (pain score at baseline, radiographic disease severity and baseline adduction moment without insoles).

## Results

Eight males (40%) and 12 females (60%) participated. Mean (SD) age, height and weight of the cohort was 63.5 (9.4) years, 1.69 (0.07) m and 83.1 (14.2) kg respectively. Eight (40%) participants demonstrated Grade 2 (mild) osteoarthritic disease on x-ray, and the remaining 12 (60%) demonstrated Grade 3 (moderate) disease. Mean (SD) WOMAC pain and physical function scores obtained were 6 (3) and 20 (14) respectively.

**Figure 2:**
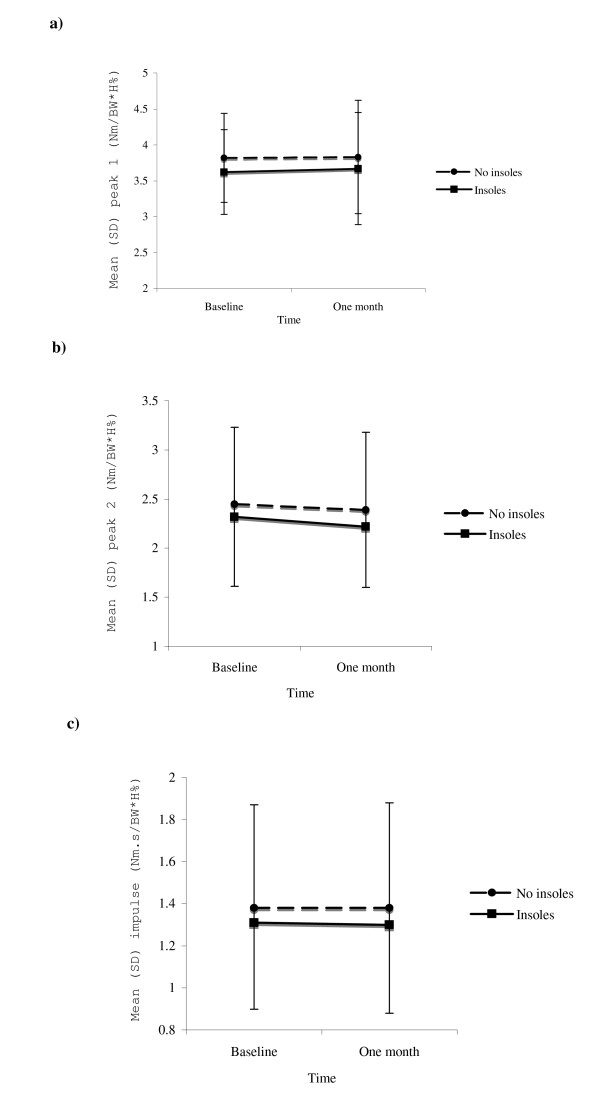
**Interaction between time and insole condition for a) first peak adduction moment; b) second peak adduction moment; and c) adduction angular impulse**.

Table 1 reports measures of the adduction moment and walking speed obtained across testing conditions at baseline and one-month follow-up. Regarding the first peak adduction moment, there was a significant main effect for condition but not for time, nor was an interaction effect evident (Fig 2a). Similar results were obtained for the second peak (Fig 2b). Regarding the adduction angular impulse, there was a significant main effect for condition but not for time, nor was an interaction effect evident (Fig 2c). As walking speed remained similar across time and condition (and no interaction effect was evident), it was not included as a covariate in data analyses.

**Table 1:** Mean (SD) knee adduction moment parameters and walking speed measured across conditions at baseline and one-month follow-up.

	Baseline			One month			p value		
	
	No insoles	Insoles	Change	No insoles	Insoles	Change	Condition	Time	Interaction
Peak 1 (Nm/BW*HT%)	3.82 (0.62)	3.62 (0.59)	-5.1%	3.83 (0.79)	3.67 (0.78)	-4.2%	<0.001	0.77	0.63
Peak 2 (Nm/BW*HT%)	2.45 (0.78)	2.32 (0.84)	-7.0%	2.39 (0.79)	2.22 (0.79)	-7.4%	<0.001	0.30	0.63
Impulse (Nm.s/BW*HT%)	1.38 (0.49)	1.31 (0.48)	-5.2%	1.38 (0.50)	1.30 (0.50)	-6.7%	<0.001	0.85	0.59
**Walking speed (m/s)**	1.27 (0.23)	1.27 (0.21)	0.6%	1.27 (0.22)	1.27 (0.24)	-0.1%	0.94	0.75	0.98

Peak 1 = peak adduction moment in the first half of stance; Peak 2 = peak adduction moment in the second half of stance

Change calculated as the score obtained without insoles subtracted from the score obtained with insoles and divided by the score obtained without insoles and expressed as a percentage

**Figure 3:**
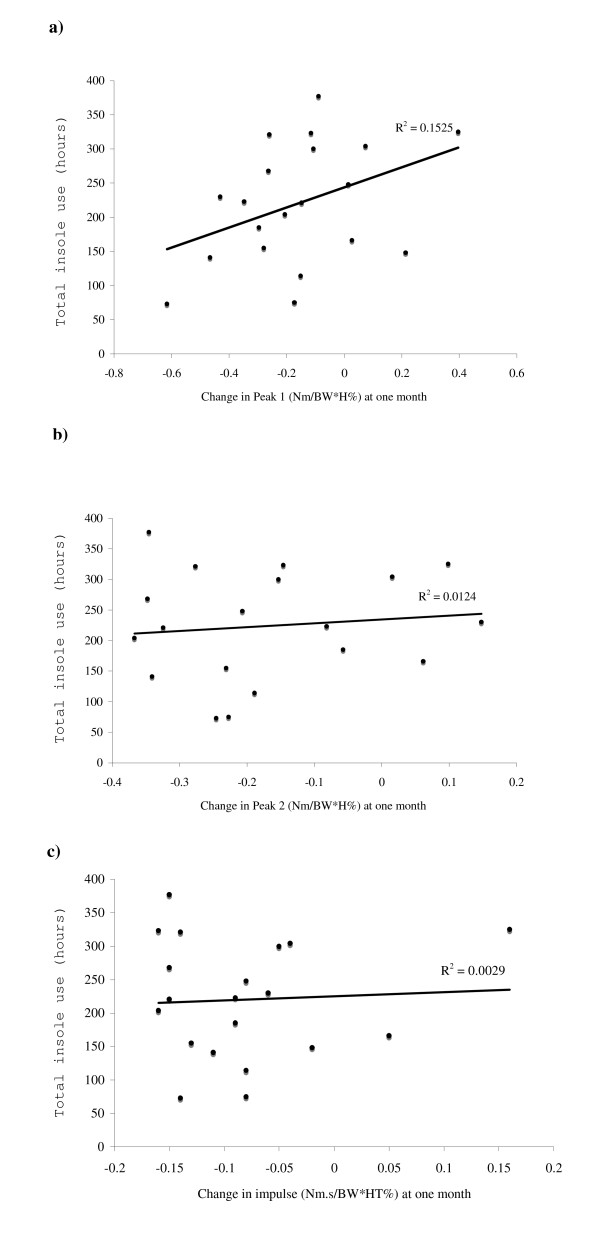
**Scatterplots depicting relationships between total insole use and change in a) first peak adduction moment; b) second peak adduction moment; and c) adduction angular impulse with insoles at one month**.

Participants wore the insoles for a mean (SD) of 46 (24) hours in the first week, and for 61 (25), 57 (28) and 57 (27) hours in weeks 2, 3 and 4 respectively. Over the four- week period, insoles were worn for a mean (SD) total time of 220 (88) hours. There was no relationship between total insole use and change in the first peak adduction moment (r = 0.39, p = 0.09), the second peak (r = 0.11, p = 0.65) or the adduction angular impulse (r = -0.06, p = 0.80) with insoles at follow-up (Fig 3). There was no difference in mean change in either the first peak or second peak adduction moment or the adduction angular impulse with insoles at follow-up between high and low users of insoles (-0.11 vs -0.21 Nm/BW*H%, p = 0.122; -0.13 vs -0.21 Nm/BW*H%, p = 0.33 and; -0.10 vs -0.07 Nm.s/BW*HT%, p = 0.78 respectively).

## Discussion

Laterally wedged insoles are advocated for the management of medial tibiofemoral knee OA [5,6] and biomechanical data have shown that such an intervention can immediately reduce the knee adduction moment by 5-10% on average [7-10]. No study to date has evaluated whether the biomechanical effects of lateral wedges decline over time. This study evaluated whether the effect of laterally wedged insoles on the adduction moment declined over one month of wear. Results revealed that the immediate beneficial effects of the insole on the adduction moment remained even after the insoles had been worn by participants on a daily basis for one month. Furthermore, high users of the insoles demonstrated similar reductions in adduction moment parameters at one month to participants who were low users of the insoles over the preceding four weeks.

This is the first study to evaluate change in the biomechanical effects of laterally wedged insoles over any timeframe. Previous studies in knee OA have evaluated the immediate effects of insoles only, thus there are no similar studies with which to compare our results. Contrary to expectations, effects of the insoles on the adduction moment did not decline over one month. A limitation of this study is the sample size of 20 participants, and although we detected significant main effects, it is possible that there were insufficient participants to detect a significant interaction. It is also quite possible that a decline in the biomechanical benefits of wedges could be seen after a longer timeframe. As we did not objectively measure the degree to which our participant's wedges had compressed or worn down, it is impossible to know whether the insoles in this study did in fact degrade over such a relatively short period. Nonetheless, participants reported an average total insole use of 222 hours over four weeks, indicating use of insoles for approximately 8 hours each day. These data suggest that participants were wearing the insoles as prescribed and it is reasonable to assume that some compression/degradation would occur over a one-month period.

From a clinical perspective, findings from this study suggest that the biomechanical effectiveness of laterally wedged insoles does not decline after one month of wear. However the sample size of this study was limited to 20 participants and further studies of larger samples are needed to validate the current findings. While it is not presently known how frequently insoles need to be changed in order to optimise outcome, this study suggests that patients can continue to wear their insoles for periods greater than one month. Future research should serially evaluate the effects of insoles over longer periods of time, to more precisely ascertain exactly when biomechanical effects begin to decline and to guide health practitioners as to how frequently new insoles should be provided. It is also important to recognise that laterally wedged insoles are not always inserted into shoes, but may also be worn without shoes via strapping to the foot or within a sock-type ankle supporter [21,22]. It is possible that results of the current study may differ if other types of lateral wedges are used and further research is needed to determine if this is the case.

Although consistent with other studies, the reductions in the adduction moment observed with lateral wedged insoles in this study (4.2-5.1%) are marginally smaller than the reductions reported by us [10] and others [7,8] and somewhat smaller than the 8.7% reduction reported by Butler et al [9]. It is not clear why this is the case but differences in the design of wedges and the footwear utilised across studies may explain variations in results. For example, in comparison to our study, Butler et al [9] customised the degree of lateral wedging for each participant in order to achieve maximal pain relief during a step-down task. A mean of 9.6° (+/- 3.2) of wedging was used, which may explain the greater reductions in the adduction moment observed by these authors compared to us.

Symptomatic response to insoles was not evaluated in this study, thus it should not be assumed that clinical benefits of insoles also remain after one month of use. While it is believed that alleviation of knee pain occurs in response to the reduction in medial compartment load observed with laterally wedged insoles [23], results from clinical trials remain inconclusive about the benefits of insoles on symptoms [24]. Future research should also evaluate whether any symptomatic benefit with insoles declines over time as insoles undergo continuous wear, as it is possible that decline in symptomatic benefit (if present) occurs at a rate different to decline in biomechanical benefits.

## Conclusion

In summary, findings from this study show that the load-reducing effects of laterally wedged insoles on the adduction moment do not appear to decline after one month of continuous use, suggesting that significant wedge degradation does not occur over the short-term.

## Competing interests

The authors declare that they have no competing interests.

## Authors' contributions

RSH & KLB conceived of the study, designed the study and obtained funding. KAB recruited study participants and performed data collection and entry. RSH performed statistical analyses and drafted the manuscript. KLB & KAB contributed to the draft manuscript. All authors read and approved the final manuscript.

## Pre-publication history

The pre-publication history for this paper can be accessed here:

http://www.biomedcentral.com/1471-2474/10/146/prepub
